# In Silico Docking and In Vitro Approaches towards BACE1 and Cholinesterases Inhibitory Effect of Citrus Flavanones

**DOI:** 10.3390/molecules23071509

**Published:** 2018-06-22

**Authors:** Seungeun Lee, Kumju Youn, GyuTae Lim, Jinhyuk Lee, Mira Jun

**Affiliations:** 1 Department of Food Science and Nutrition, Dong-A University, 37, Nakdong-daero 550 beon-gil, Saha-gu, Busan 49315, Korea; lse2340@naver.com (S.L.); kjyoun@dau.ac.kr (K.Y.); 2 Korean Bioinformation Center, Korea Research Institute of Bioscience and Biotechnology (KRIBB), 125, Gwahak-ro, Yuseong-gu, Daejeon 34141, Korea; gyutae@kribb.re.kr (G.L.); jinhyuk@kribb.re.kr (J.L.); 3 Department of Bioinformatics, KIRBB School of Bioscience, Korea University of Sciences and Technology, 217 Gajung-ro, Yuseong-gu, Daejeon 34113, Korea

**Keywords:** Alzheimer’s disease, BACE1, cholinesterases, in silico docking, citrus flavanones, hesperidin

## Abstract

Alzheimer’s disease (AD) is the most prevalent neurodegenerative disease, distinctively characterized by senile plaques, neurofibrillary tangles, and synaptic loss, finally resulting in neuronal death. β-Site amyloid precursor protein (APP) cleaving enzyme 1 (BACE1) and cholinesterases have been identified as therapeutic targets for AD, and the discovery of their inhibitors is of critical importance for developing preventive strategies for AD. To discover natural multi-target compounds possessing BACE1, acetylcholinesterase (AChE), and butyrylcholinesterase (BChE) inhibitory properties, major citrus flavanones including hesperetin, naringenin, and hesperidin were evaluated. In vitro anti-AD activities were performed via BACE1 and cholinesterases inhibition assays, as well as enzyme kinetic predictions. For the design of potential inhibitors of AD-related enzymes, molecular docking analysis was performed. Based on the biological evaluation, hesperidin demonstrated the best inhibitory properties toward BACE1, AChE, and BChE, with IC_50_ values of 10.02 ± 1.12, 22.80 ± 2.78, and 48.09 ± 0.74 µM, respectively. Kinetic studies revealed that all tested compounds were found to be noncompetitive inhibitors against BACE1 and cholineseterases. In addition, molecular docking studies of these compounds demonstrated negative binding energies for BACE1, AChE, and BChE, indicating high affinity and tight binding capacity for the target enzymes. The present study suggested that the selected citrus flavanones could act together as multiple inhibitors of BACE1, AChE, and BChE, indicating preventive and therapeutic potential against AD.

## 1. Introduction

Alzheimer′s disease (AD), characterized by the appearance of senile plaques and neurofibrillary tangles, as well as a loss of cholinergic neurons, is the most prevalent form of dementia [1]. Senile plaques contain a major protein known as the β-amyloid protein (Aβ), whereas neurofibrillary tangles are insoluble twisted fibers inside nerve cells, consisting of hyper-phosphorylated tau protein [2]. Over the years, the “amyloid cascade hypothesis” has emerged as the principal mechanism of AD pathology, with in vivo evidence having demonstrated that aggregated Aβ induces neurofibrillary tangle formation as well as neuronal death [3,4].

Aβ peptide is generated by sequential cleavage of amyloid precursor protein (APP) by β- and γ-secretase. Studies show that β-secretase (β-site amyloid precursor protein cleaving enzyme 1, BACE1) protein levels and activity are elevated in sporadic AD brains, and that BACE1 levels are upregulated under stress conditions such as oxidative stress, cerebral ischemia, and hypoxia, all of which are associated with increased AD incidence [5,6,7]. Given that BACE1 is the initial and rate-limiting step in Aβ production, it is considered a prime target for the treatment and prevention of AD. 

In addition to BACE1, the cholinergic hypothesis has also played a large part in the development of AD therapy. The neurotransmitter acetylcholine (ACh) possesses an important role in the process of learning and memory in the hippocampus. Under normal physiological conditions, acetylcholinesterase (AChE) is the major enzyme carrying out the hydrolysis of ACh into choline and acetate, whereas butyrylcholinesterase (BChE) acts as a co-regulator of the activity of AChE [8]. However, during the development of AD, AChE activity decreases in the temporal cortex and hippocampus, while BChE activity increases, compensating for some of the functions of AChE in cholinergic neurons [9]. Besides playing a role in the hydrolysis of ACh, both enzymes also possess nonenzymatic functions, in which they are found to associate with Aβ aggregation and neurofibrillary tangles in mouse and human AD brain [9,10,11]. In addition, both AChE and BuChE are related to inflammatory pathways through increasing cytokine levels in the AD brain [12]. Therefore, inhibition of both enzymes is a highly desirable feature of AD therapy. 

In citrus fruits, flavanones comprise approximately 95% of the total citrus flavonoids, existing in both aglycone and glycosidic forms. The most abundant flavanone aglycones are hesperetin and naringenin. Hesperidin is the major glycoside with rutinose (rhamnosyl-α-1,2 glucose) [13]. Various studies on the biological effects of these compounds have reported that they possess anti-inflammatory, anti-oxidant, anti-mutagenic, and anti-carcinogenic activities [14,15]. Furthermore, the main citrus flavanones have been observed to exhibit neuroprotective effects against Aβ, oxidative stress, and neuroinflammation in several in vitro and in vivo studies [16,17,18]. Although some studies have reported the neuroprotective properties of hesperetin, naringenin, and hesperidin, their direct effects on BACE1, along with AChE and BChE, have not been fully evaluated. In our previous study, polymethoxyflavones from citrus peel significantly inhibited BACE1 activity, leading us to study citrus flavanones as AD-related enzyme inhibitors. The present study focused on potent inhibition by hesperetin, naringenin, and hesperidin by evaluating enzyme activities, enzyme kinetics, and in silico docking simulation predictions, potentially targeting multiple pathological routes of AD.

## 2. Results

### 2.1. Inhibiting Multiple Enzyme Targets of Hesperetin, Naringenin, and Hesperidin

**Figure 1:**
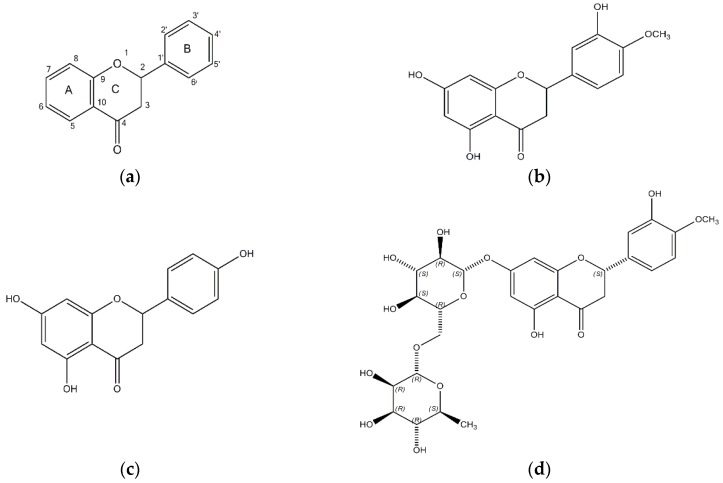
The chemical structures of (**a**) flavanone; (**b**) hesperetin; (**c**) naringenin; and (**d**) hesperidin.

The structures of hesperetin (4′-methoxy-3′,5,7-trihydroxyflavanone), naringenin (4,5,7-trihydroxyflavanone), and hesperidin are shown in Figure 1. As shown in Table 1, hesperidin exhibited the most potent inhibitory action on BACE1 (IC_50_, 16.99 ± 1.25 µM), followed by hesperetin (IC_50_, 22.13 ± 1.81 µM) and naringenin (IC_50_, 30.31 ± 2.06 µM). In addition, the IC_50_ value of hesperidin was similar to that of resveratrol, which was used as a positive control. 

The potential causes in AD is the Aβ, which is generated through sequential cleavage by β- and γ-secretase. Cleavage by α-secretase produces a soluble N-terminal fragment, APPsα, and a membrane-bound C-terminal fragment APP (C83). The remaining C83 is then cleaved by γ-secretase to release a nontoxic p3, thereby precluding Aβ generation. Tumor necrosis-α converting enzyme (TACE) is a candidate of α-secretases belonging to the ADAM (a disintegrin and metalloprotease) family. To determine the BACE1 selectivity of citrus flavanones, all compounds were tested against TACE, trypsin, chymotrypsin, and elastase, and found to be inactive at up to 100 µM (Table 2). 

Hesperidin exerted a strong inhibitory effect on AChE and BChE, with IC_50_ values of 22.80 ± 2.78 µM and 48.09 ± 0.74 µM, respectively. However, the inhibitory potential of hesperetin and naringenin was relatively specific to AChE, with IC_50_ values of 45.70 ± 2.69 and 42.66 ± 4.30 µM, but not BChE.

### 2.2. Enzyme Kinetics of Hesperetin, Naringenin, and Hesperidin

**Figure 2:**
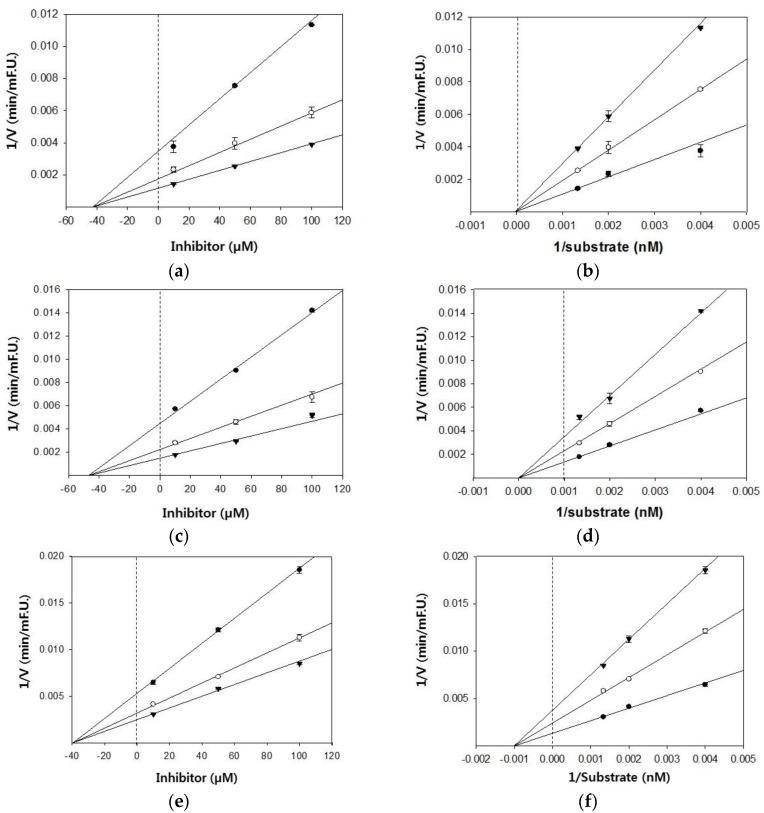
Dixon and Lineweaver–Burk plots for the inhibition of BACE1 by citrus flavanones. Dixon plots show the effects of the presence of different substrate concentrations: 250 nM (●), 500 nM (○), 750 nM (▼) for hesperetin (**a**); naringenin (**c**); and hesperidin (**e**). Lineweaver–Burk plots were analyzed in the presence of different inhibitor concentrations: 10 μM (●), 50 μM (○), 100 μM (▼) for hesperetin (**b**); naringenin (**d**); and hesperidin (**f**).

**Figure 3:**
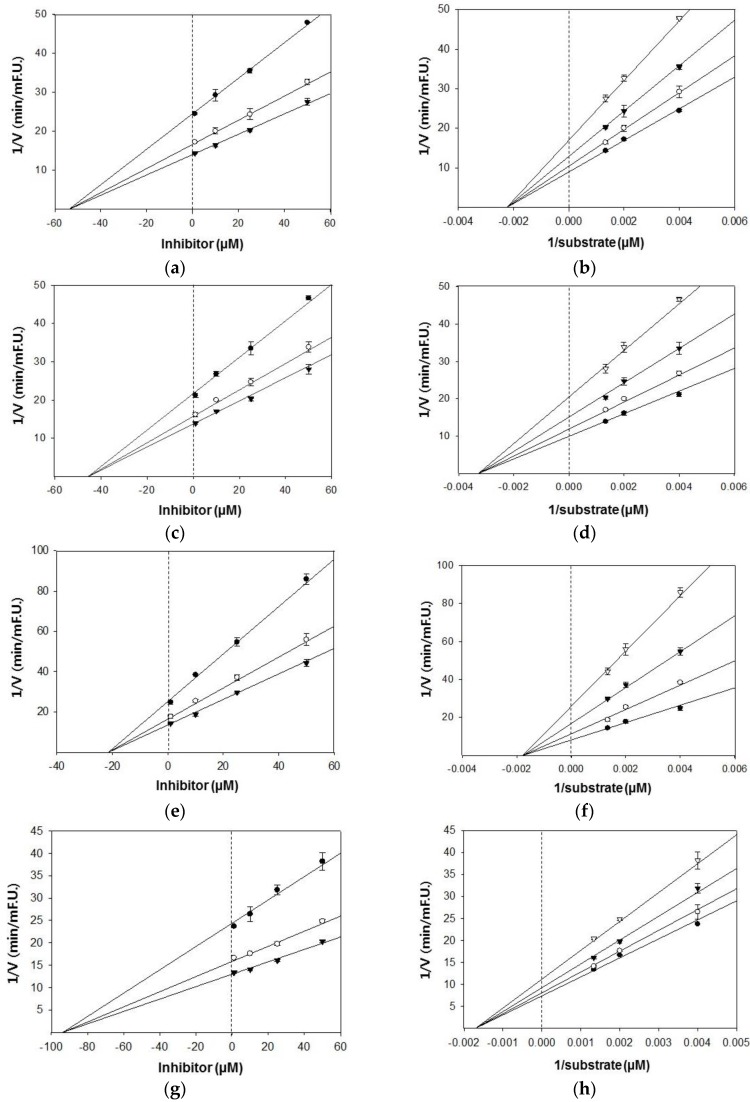
Dixon and Lineweaver–Burk plots for the inhibition of AChE by citrus flavanones. Dixon plots show the effects of the presence of different substrate concentrations: 250 nM (●), 500 nM (○), 750 nM (▼) for hesperetin (**a**); naringenin (**c**); and hesperidin (**e**). Lineweaver–Burk plots were analyzed in the presence of different inhibitor concentrations: 1 μM (●), 10 μM (○), 25 μM (▼), 50 μM (▽) for hesperetin (**b**); naringenin (**d**); and hesperidin (**f**). Dixon and Lineweaver–Burk plots for the inhibition of BChE by hesperidin. Dixon plots show the effects of the presence of different substrate concentrations: 250 nM (●), 500 nM (○), 750 nM (▼) for hesperidin (**g**). Lineweaver–Burk plots were analyzed in the presence of different inhibitor concentrations: 1 μM (●), 10 μM (○), 25 μM (▼), 50 μM (▽) for hesperidin (**h**).

Based on the IC_50_ values, the inhibition modes and parameters of the three tested compounds on the targeted enzymes were investigated. The results indicated that the compounds noncompetitively inhibited BACE1 and cholinesterases. The Ki values for the inhibition of BACE1 by hesperidin, naringenin, and hesperetin were 39.4 μM, 47.1 μM, and 42.7 μM, respectively (Figure 2). Because lower Ki values denote tighter binding with the enzyme, hesperidin was determined to be the most effective BACE1 inhibitor. Likewise, hesperidin was the strongest inhibitor of AChE and BChE, with Ki values of 21.4 and 93.6 μM, respectively (Figure 3). Hesperetin and naringenin exerted inhibition towards AChE with Ki values of 53.9 and 45.4 μM, respectively.

### 2.3. Molecular Docking Study of the Inhibitory Activity of Major Citrus Flavanones

**Figure 4:**
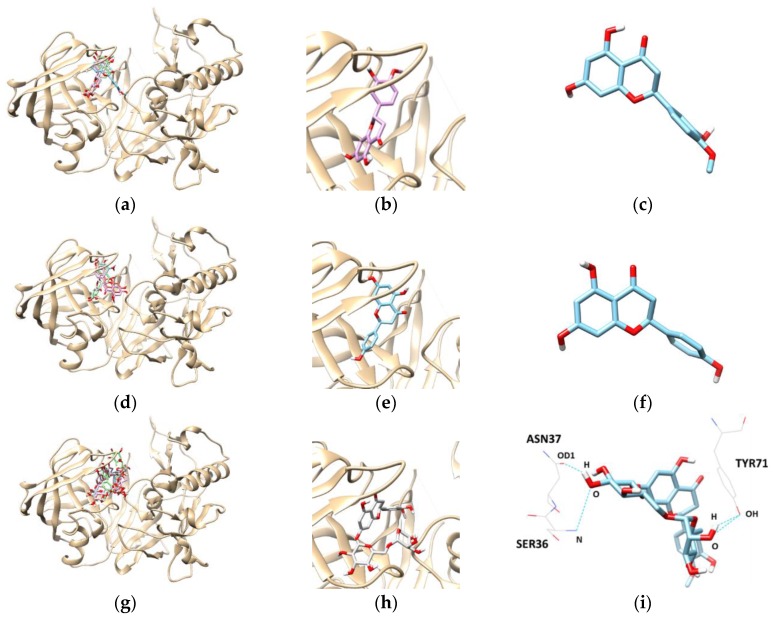
The best docked poses between BACE1 and hesperetin (**a**); naringenin (**d**); and hesperidin (**g**). View of the binding site magnified from hesperetin (**b**); naringenin (**e**); and hesperidin (**h**). Hydrogen interaction diagram of hesperetin (**c**); naringenin (**f**); and hesperidin (**i**). The blue dotted lines indicate hydrogen bonds between flavanones and BACE1.

To get an insight into the molecular interactions between the compounds and targeted enzymes, a binding analysis was conducted using the Autodock Vina docking program. The lowest binding energy, the number of hydrogen bonds, and interacting residues with BACE1 are listed in Table 3 and Figure 4. The results demonstrated that the lowest binding energies of hesperetin, naringenin, and hesperidin were −8.3, −8.1, and −10.10 kcal/mol, respectively. Lower the binding energy, greater is the binding capacity of the ligand, suggesting that BACE1 binds and reacts preferentially with hesperidin. Furthermore, hesperidin is bound with the SER36, TYR71, and ASN37 residues of BACE1, connected by three hydrogen bonds with bonding distances of 3.372, 2.837, and 2.820 Å, respectively, whereas the other compounds did not form hydrogen bonds. 

**Figure 5:**
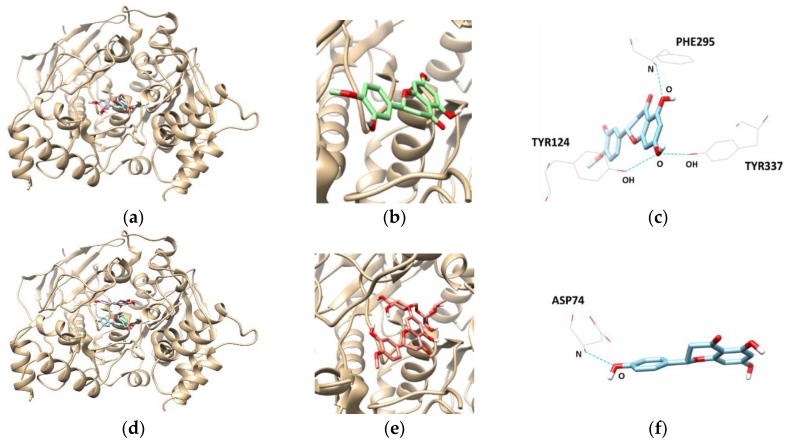

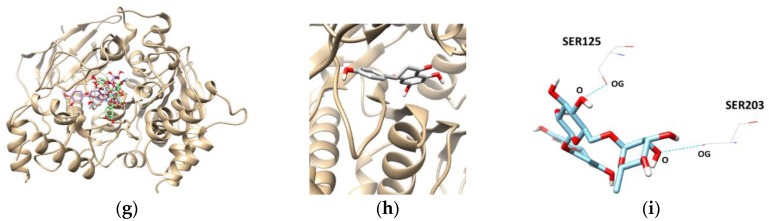
The best docked poses between AChE and hesperetin (**a**); naringenin (**d**); and hesperidin (**g**). View of the binding site magnified from hesperetin (**b**); naringenin (**e**); and hesperidin (**h**). Hydrogen interaction diagram of hesperetin (**c**); naringenin (**f**); and hesperidin (**i**). The blue dotted lines indicate hydrogen bonds between flavanones and AChE.

Docking interactions between tested flavanones and AChE were shown in Table 4 and Figure 5. Two hydrogen bonds were found between hesperidin and the AChE residues, with a binding affinity of −9.8 kcal/mol. In addition, SER125 and SER203 participated in hydrogen-interactions with bond distances of 2.810 Å and 3.094 Å, respectively. The naringenin–AChE complex (lowest energy: −8.7 kcal/mol) was stabilized by the formation of a hydrogen bond between residue ASP74 and the hydroxyl group of naringenin at a distance of 2.738 Å. Similarly, the complex of AChE and hesperetin showed the lowest binding energy of −8.4 kcal/mol, and formed three hydrogen bonds at TYR124, PHE295, and TYR337. 

**Figure 6:**
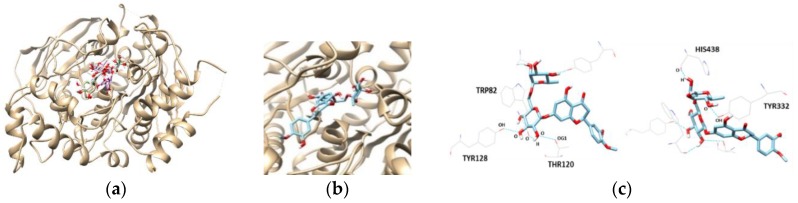
The best docked poses between BChE and hesperetin (**a**).View of the binding site magnified from hesperidin (**b**). Hydrogen interaction diagram of hesperidin (**c**). The blue dotted lines indicate hydrogen bonds between flavanones and BChE.

The most promising inhibitor of BChE, hesperidin, was subjected to a docking study. Hesperidin showed a binding affinity of −10.3 kcal/mol, as shown in Table 4 and Figure 6. In addition, the THR120, TYR128, TYR332, TRP82, and HIS438 residues were separately involved in five hydrogen bond interactions, with the oxygen atoms present in the sugar molecules of hesperidin. Interestingly, the docking scores and binding interactions of the three flavanones correlated with their inhibitory activities against AChE and BChE.

## 3. Discussion

In the present study, the three citrus flavanones were investigated for their inhibitory activities on BACE1, AChE, and BChE. However, the results in our study showed that hesperidin, containing two sugar moieties, exhibited notable BACE1 and cholinesterases-inhibitory activity, when compared with hesperetin and naringenin. Similar results regarding the correlation between sugar moieties and AChE inhibitory activity was shown in a previous study [19]. Rubrofusarin -6-*O*-β-d-gentiobioside, possessing two sugar moieties, showed more potent activity against AChE, with an IC_50_ of 15.94 ± 0.32 µM, than its aglycone form (IC_50_ > 100 µM). On the other hand, several studies reported that hesperidin and hesperetin exhibited low inhibitory activity against cholinesterases [20,21].

Docking results demonstrated that the lowest binding energies of glycosylated flavanone with the target enzyme including BACE1, AChE, and BChE were generally lower than that of the aglycone. Hesperidin probably increased the hydrophilicity of hesperetin and naringenin, thereby raising hydrogen interactions between hesperidin and its target proteins. The result of hesperidin is in accordance with the finding reported by Remya et al., that hesperidin has low binding energy with AChE [22].

Hesperidin, a glycosidic compound has previously exhibited potent neuroprotective activity in vivo. Li and coworkers (2015) reported that oral administration of hesperidin (100 mg/kg) for 10 days recovered microglial activation, APP expression, Aβ deposition, and behavioral dysfunction in APP/PS1 Tg mice [16]. In addition, chronic administration of this compound for 16 weeks attenuated oxidative damage, increased anti-oxidative defense, and alleviated cognitive impairment in APPswe/PS1dE9 Tg mice [18]. Pre-administration of hesperetin (40 mg/kg, oral) significantly attenuated cadmium-induced oxidative stress and mitochondrial dysfunction, restored antioxidant and membrane-bound enzyme activities, and decreased apoptosis in the brains of rats. Heo et al. reported that when naringenin was orally administered at 4.5 mg/kg body weight of mice, it significantly mitigated scopolamine-induced amnesia [23]. 

To develop functional foods and dietary supplements preventing or treating AD, oral bioavailability, metabolic transformation, and the blood–brain barrier (BBB) permeability of active components is a cause of concern. When absorbed, hesperidin is hydrolyzed by the gut microflora into hesperetin, and conjugated by phase II drug-metabolizing enzymes into glucuronidated, sulfated, or sulfoglucuronidated forms [24]. Hesperetin-7-*O*-glucuronide (GLN) and hesperetin-3′-*O*-GLN were identified as the predominant hesperidin metabolites. In addition, hesperetin-5,7-,3′,7-*O*-diglucuronide, hesperetin-7-*O*-glucoside, and hesperetin-3′-*O*-sulphate were also found [25]. The absorption of hesperetin and naringenin was much faster than that of hesperidin, after oral administration, reflecting the omission of the hydrolytic step. As major metabolites of naringenin, naringenin-7-*O*-GLN and naringenin-3′-*O*-GLN were detected. In general, metabolites of flavonoids showed reduced bioactivity compared to their parent compounds, though contrary results have also been reported. Hesperetin metabolites derived from hesperetin-administered rat serum were revealed to be potent antioxidants, and possess anti-inflammatory activity, when compared with hesperidin or hesperetin [26]. 

For central nervous system (CNS) penetration, the compounds must first cross the BBB, which ultimately controls the composition of extracellular fluid in the CNS, by firmly regulating molecular traffic and buffering against changes in the systemic circulation. An in vitro study by Youdim et al. discovered that naringenin and hesperetin cross the BBB in the ECV304/C6 co-culture model [27]. Peng and co-workers have identified both naringenin and its glucuronide in the cerebral cortexes of rats, following intravenous administration of naringenin (20 mg/kg), thus avoiding metabolism in the gastrointestinal tract [28]. Hesperetin was administered intravenously (50 mg/kg) and detected in the striatum of rat brains [29]. In addition, after systemic administration of hesperidin to rats, the presence of hesperidin was confirmed by brain homogenates [30]. After oral administration of hesperidin, hesperetin was detected in the rat brain [31]. The above results suggest that our tested compounds penetrate the BBB and may act directly in their intact forms on the brain. Recently, Kheradmand and coworkers (2018) reported that hesperetin nanoparticles improved therapeutic potential for AD by promoting their permeability into hippocampal neurons as well as by oral absorption [32]. Collectively, these results suggest that these citrus flavanones are promising neuroprotective candidates.

## 4. Materials and Methods 

### 4.1. Chemicals and Reagents

Hesperetin (>95% purity), naringenin (>95% purity), hesperidin (>97% purity), resveratrol (>99% purity), galanthamine, AChE, BChE, 5,5′-dithiobis-(2 nitrobenzoic acid) (DTNB), and acetylthiocholine iodide were purchased from Sigma-Aldrich (St. Louis, MO, USA). The BACE1 kit from Invitrogen (Pan Vera, Madison, WI, USA) was used for the measurement of BACE1 activity. α-Secretase (tumor necrosis factor-α converting enzyme, TACE) and its fluorogenic substrate were purchased from R&D Systems (Minneapolis, MN, USA). Elastase, chymotrypsin, trypsin, and their substrates were obtained from Sigma-Aldrich.

### 4.2. Enzymatic Assessment for Biological Evaluation

BACE1, TACE, chymotrypsin, trypsin, and elastase assays were carried out according to previously described methods [33]. BACE1, TACE, trypsin, chymotrypsin, and elastase assays were carried out using Rh-EVNLDAEFK-Quencher, Mca-PLAQAV-Dpa-RSSSR-NH_2_, *N*-benzoyl-l-Arg-pNA, *N*-benzoyl-l-Tyr-pNA, and *N*-succinyl-Ala-Ala-Ala-pNA as substrates, respectively. 

AChE and BChE inhibitory activities were assessed by a modified version of the Ellman colorimetric method [34]. Each reaction mixture consisted of sodium phosphate buffer (pH 7.4), test sample solution, and either AChE (electrophorus electricus, 0.8 U/mL) or BChE (equine serum, 0.8 U/mL) solution, which was then mixed and incubated for 15 min at room temperature. Reactions were initiated upon addition of DTNB and substrate (0.75 mM). 

Enzymatic hydrolysis, mediated by AChE or BChE, was monitored according to the formation of yellow 5-thio-2-nitrobenzoate anions at 405 nm for 15 min, which were produced by the reaction of DTNB with thiocholine released from ACh or BCh. All reactions were performed in 96-well plates in triplicate, and recorded using a microplate spectrophotometer (Molecular Devices, Sunnyvale, CA, USA).

### 4.3. Assessment of Inhibition Kinetics on BACE1, AChE, and BChE

To prove the kinetic mechanisms of hesperetin, naringenin, and hesperidin towards BACE1, AChE, and BChE, both Dixon plots and Lineweaver–Burk complementary kinetic methods were conducted. The inhibitory constant (Ki) was obtained by interpretation of the Dixon plot, and Vmax and Km were defined by Lineweaver–Burk plots, using the initial velocities obtained over a substrate concentration range. These kinetic parameters were calculated using the Enzyme Kinetic™ module of SigmaPlot™ version 12.3 (Systat Software, Inc., San Jose, CA, USA).

### 4.4. Molecular Docking Studies of BACE1, AChE, and BChE

The Autodock Vina program was used for molecular docking study to investigate structural complexes of the BACE1, AChE, and BChE with flavanones. The target proteins, BACE1, AChE, and BChE were obtained from the Protein Data Bank (PDB, http://www.rcsb.org/), with the respective accession codes 2WJO, 4PQE, and 1P0I, respectively [35,36,37]. The Autodock Vina parameters, the dimension of grid: 30 × 30 × 30 Å, cluster radius: 1 Å, the Cα coordinates in each selected backbone binding residues of protein receptor were used for the center of docking space. Other options for docking simulations were used as defaults. The atomic coordinates of the ligands were drawn and displayed using Marvin sketch (5.11.4, 2012, ChemAxon, One Broadway Cambridge, MA, USA). The calculated geometries were ranked in terms of free energy of binding, the lowest energy, and the best poses were selected. 

### 4.5. Statistics

All results were presented as the mean ± SD of three independent experiments. Statistical significance was assessed by Duncan′s multiple range tests using Statistical Analysis System (SAS) version 9.3 (SAS Institute, Cary, NC, USA).

## 5. Conclusions

The present study suggests that hesperetin, naringenin, and hesperidin show noncompetitive inhibition against BACE1 and cholinesterases. In our docking study, hesperidin with hydroxyl groups of the sugar moiety, available for hydrogen bonding with amino-acid residues in target enzymes, were most effective. Although further in vivo studies of the tested compounds are required in order to confirm our present findings, these citrus flavanones might be useful as potentially functional compounds for the treatment and prevention of AD.

## Figures and Tables

**Table 1:** Inhibitory activities of hesperetin, naringenin, and hesperidin on BACE1 and cholinesterases.

Compounds	IC_50_ (µM) ^1^		
	BACE1	AChE	BChE
Hesperetin	22.13 ± 1.81	45.70 ± 2.69	>100
Naringenin	30.31 ± 2.06	42.66 ± 4.30	>100
Hesperidin	16.99 ± 1.25	22.80 ± 2.78	48.09 ± 0.74
Resveratrol ^2^	14.59 ± 0.79	-	-
Galanthamine ^3^	-	1.59 ± 0.03	10.93 ± 0.69

^1^ The 50% inhibition concentration (µM) is expressed as mean ± S.E. of triplicate experiments. ^2^ Resveratrol and ^3^ Galanthamine were used as positive reference controls in the BACE1 and cholinesterases assays, respectively.

**Table 2:** Inhibitory activities (%) ^1^ of hesperetin, naringenin, and hesperidin against α-secretase (tumor necrosis factor-α converting enzyme, TACE) and other serine proteases.

Sample (μM)	TACE	Trypsin	Chymotrypsin	Elastase
Hesperetin				
10	8.66 ± 0.70	5.31 ± 0.39	4.91 ± 1.40	4.84 ± 0.46
100	11.31 ± 1.37	4.72 ± 0.45	9.21 ± 0.33	9.95 ± 1.28
Naringenin				
10	5.15 ± 0.98	7.80 ± 1.25	8.77 ± 0.74	9.74 ± 0.41
100	6.95 ± 1.52	5.84 ± 0.75	6.88 ± 0.89	10.51 ± 1.17
Hesperidin				
10	8.53 ± 1.05	2.27 ± 0.30	3.19 ± 0.28	6.91 ± 0.83
100	9.19 ± 1.20	1.41 ± 0.22	4.11 ± 0.34	6.00 ± 1.33

^1^ The inhibitory activity (%) is expressed as mean ± S.E. of triplicate experiments.

**Table 3:** Lowest energies and the number of hydrogen bond interactions of citrus flavanones in BACE1.

Enzyme	Ligands	Lowest Energy (Kcal/mol)	H-Bonds No.	Residues	Bond Distance (Å)
BACE1	Hesperetin	−8.3	-	-	-
	Naringenin	−8.1	-	-	-
	Hesperidin	−10.1	3	SER36 TYR71 ASN37	3.372 2.837 2.820

**Table 4:** Lowest energies and the number of hydrogen bond interactions of citrus flavanones in cholinesterases.

Enzyme	Ligands	Lowest Energy (Kcal/mol)	H-Bonds No.	Residues	Bond Distance (Å)
AChE	Hesperetin	−8.4	3	TYR124 PHE295 TYR337	2.927 2.913 2.699
	Naringenin	−8.7	1	ASP74	2.738
	Hesperidin	−9.8	2	SER125 SER203	2.810 3.094
BChE	Hesperidin	−10.3	5	THR120 TYR128 TRP82 HIS438 TYR332	2.929 2.696 3.366 2.617 3.006

## References

[1] Anand R., Gill K.D., Mahdi A.A. (2014). Therapeutics of Alzheimer’s disease: Past, present and future. Neuropharmacology.

[2] Walsh D.M., Selkoe D.J. (2007). Aβ Oligomers—A decade of discovery. J. Neurochem..

[3] Götz J., Schild A., Hoerndli F., Pennanen L. (2004). Amyloid-induced neurofibrillary tangle formation in Alzheimer’s disease: Insight from transgenic mouse and tissue-culture models. Int. J. Dev. Neurosci..

[4] Cole S.L., Vassar R. (2007). The Alzheimer’s disease β-secretase enzyme, BACE1. Mol. Neurodegener..

[5] Tamagno E., Bardini P., Obbili A., Vitali A., Borghi R., Zaccheo D., Pronzato M.A., Danni O., Smith M.A., Perry G. (2002). Oxidative stress increases expression and activity of BACE in NT2 neurons. Neurobiol. Dis..

[6] Wen Y., Onyewuchi O., Yang S., Liu R., Simpkins J.W. (2004). Increased β-secretase activity and expression in rats following transient cerebral ischemia. Brain Res..

[7] Zhang X., Zhou K., Wang R., Cui J., Lipton S.A., Liao F.F., Xu H., Zhang Y.W. (2007). Hypoxia-inducible factor 1α (HIF-1α)-mediated hypoxia increases BACE1 expression and β-amyloid generation. J. Biol. Chem..

[8] Bhakta H.K., Park C.H., Yokozawa T., Tanaka T., Jung H.A., Choi J.S. (2017). Potential anti-cholinesterase and β-site amyloid precursor protein cleaving enzyme 1 inhibitory activities of cornuside and gallotannins from *Cornus officinalis* fruits. Arch. Pharm. Res..

[9] Ciro A., Park J., Burkhard G., Yan N., Geula C. (2012). Biochemical differentiation of cholinesterases from normal and Alzheimer’s disease cortex. Curr. Alzheimer Res..

[10] Inestrosa N.C., Alvarez A.C., Pérez A., Moreno R.D., Vicente M., Linker C., Casanueva O.I., Soto C., Garrido J. (1996). Acetylcholinesterase accelerates assembly of amyloid-beta-peptides into Alzheimer’s fibrils: Possible role of the peripheral site of the enzyme. Neuron.

[11] Darvesh S., Hopkins D.A., Geula C. (2003). Neurobiology of butyrylcholinesterase. Nat. Rev. Neurosci..

[12] Das U.N. (2007). Acetylcholinesterase and butyrylcholinesterase as possible markers of low-grade systemic inflammation. Med. Sci. Monit..

[13] Cirmi S., Ferlazzo N., Lombardo G.E., Ventura-Spagnolo E., Gangemi S., Calapai G., Navarra M. (2016). Neurodegerative disease: Might citrus flavonoids play a protective role?. Molecules.

[14] Nalini N., Aranganathan S., Kabalimurthy J. (2012). Chemopreventive efficacy of hesperetin (citrus flavonone) against 1, 2-dimethylhydrazine-induced rat colon carcinogenesis. Toxicol. Mech. Methods.

[15] Lee J., Kim G. (2010). Evaluation of antioxidant and inhibitory activities for different subclasses flavonoids on enzymes for rheumatoid arthritis. J. Food Sci..

[16] Li C.Y., Zug C., Qu H.C., Schluesener H., Zhang Z.Y. (2015). Hesperidin ameliorates behavioral impairments and neuropathology of transgenic APP/PS1 mice. Behav. Brain Res..

[17] Huang S.M., Tsai S.Y., Lin J.A., Wu C.H., Yen G.C. (2012). Cytoprotective effects of hesperetin and hesperidin against amyloid β-induced impairment of glucose transport through downregulation of neuronal autophagy. Mol. Nutr. Food Res..

[18] Wang D.M., Liu L., Zhu X.Y., Wu W.L., Wang Y. (2014). Hesperidin alleviates cognitive impairment, mitochondrial dysfunction and oxidative stress in a mouse model of Alzheimer’s disease. Cell. Mol. Neurobiol..

[19] Shrestha S., Su S.H., Pradeep P., Jung H.A., Choi J.S. (2018). Structure related inhibition of enzyme systems in cholinesterases and BACE1 in vitro by naturally occurring naphthopyrone and its glycosides isolated from *Cassia obtusifolia*. Molecuels.

[20] Senol F.S., Ankli A., Reich E., Orhan I.E. (2016). HPTLC fingerprinting and cholinesterase inhibitory and metal-chelating capacity of various *Citrus cultivars* and *Olea europaea*. Food Technol. Biotechnol..

[21] Li B., Hunag A.-L., Zhang Y.-L., Li Z., Ding H.-W., Huang C., Meng X.-M., Li J. (2017). Design, synthesis and evaluation of hesperetin derivatives as potential multifunctional anti-Alzheimer agents. Molecules.

[22] Remya C., Dileep K.V., Tintu I., Variyar E.J., Sadasivan C. (2014). Flavanone glycosides as acetylcholinesterase inhibitors: Computational and experimental evidence. Indian J. Pharm. Sci..

[23] Heo H.J., Kim D.O., Shin S.C., Kim M.J., Kim B.G., Shin D.H. (2004). Effect of antioxidant flavanone, naringenin, from Citrus junos on neuroprotection. J. Agric. Food Chem..

[24] Matsumoto H., Ikoma Y., Sugiura M., Yano M., Hasegawa Y. (2004). Identification and quantification of the conjugated metabolites derived from orally administered hesperidin in rat plasma. J. Agric. Food Chem..

[25] Bredsdorff L., Nielsen I.L., Rasmussen S.E., Cornett C., Barron D., Bouisset F., Offord E., Williamson G. (2010). Absorption, conjugation and excretion of the flavanones, naringenin and hesperetin from a-rhamnosidase-treated orange juice in human subjects. Br. J. Nutr..

[26] Yang H.L., Chen S.C., Senthil Kumar K.J., Yu K.N., Lee Chao P.D., Tsai S.Y., Hou Y.C., Hseu Y.C. (2012). Antioxidant and anti-inflammatory potential of hesperetin metabolites obtained from hesperetin-administered rat serum: An ex vivo approach. J. Agric. Food Chem..

[27] Youdim K.A., Dobbie M.S., Kuhnle G., Proteggente A.R., Abbott N.J., Rice-Evans C. (2003). Interaction between flavonoids and the blood-brain barrier: In vitro studies. J. Neurochem..

[28] Peng H.W., Cheng F.C., Huang Y.T., Chen C.F., Tsai T.H. (1998). Determination of naringenin and its glucuronide conjugate in rat plasma and brain tissue by high-performance liquid chromatography. J. Chromatogr..

[29] Tsai T.H., Chen Y.F. (2000). Determination of unbound hesperetin in rat blood and brain by microdialysis coupled to microbore liquid chromatography. J. Food Drug Anal..

[30] Carballo-Villalobos A.I., Gonzalez-Trujano M.E., Psellicer F., López-Muñoz F.J. (2016). Antihyperalgesic Effect of Hesperidin Improves with Diosmin in Experimental Neuropathic Pain. Biomed. Res. Int..

[31] Mogami S., Sadakane C., Nahata M., Mizuhara Y., Yamada C., Hattori T., Takeda H. (2016). CRF receptor 1 antagonism and brain distribution of active components contribute to the ameliorative effect of rikkunshito on stress-induced anorexia. Sci. Rep..

[32] kheradmand E., Moghaddam A.H., Zare M. (2018). Neuroprotective effect of hesperetin and nano-hesperetin on recognition memory impairment and the elevated oxygen stress in rat model of Alzheimer’s disease. Biomed. Pharmacother..

[33] Youn K., Yu Y., Lee J., Jeong W.S., Ho C.T., Jun M. (2017). Polymethoxyflavones: Novel β-Secretase (BACE1) Inhibitors from Citrus Peels. Nutrients.

[34] Ellman G.L., Callaway E. (1961). Erythrocyte cholinesterase-levels in mental patients. Nature.

[35] Nicholls A., Mcgaughey G.B., Sheridan R.P., Good A.C., Warren G., Mathieu M., Muchmore S.W., Brown S.P., Grant J.A., Haigh J.A. (2010). Molecular Shape and Medicinal Chemistry: A Perspective. J. Med. Chem..

[36] Krátký M., Štěpánková Š., Vorčáková K., Švarcová M., Vinšová J. (2016). Novel Cholinesterase Inhibitors Based on *O*-Aromatic *N*,*N*-Disubstituted Carbamates and Thiocarbamates. Molecules.

[37] Nicolet Y., Lockridge O., Masson P., Fontecilla-Camps J.C., Nachon F. (2003). Crystal structure of human butyrylcholinesterase and of its complexes with substrate and products. J. Biol. Chem..

